# Development of an implantable three-dimensional model of a functional pathogenic multispecies biofilm to study infected wounds

**DOI:** 10.1038/s41598-022-25569-5

**Published:** 2022-12-17

**Authors:** Camila Cárdenas-Calderón, Valentina Veloso-Giménez, Tamara González, Aniela Wozniak, Patricia García, Sebastián San Martín, Juan F. Varas, Ivo Carrasco-Wong, Mario Vera, José Tomás Egaña

**Affiliations:** 1 Institute for Biological and Medical Engineering, Schools of Engineering, Medicine and Biological Sciences, Pontificia Universidad Católica de Chile, Vicuña Mackenna 4860, 7820436 Santiago, Chile; 2 Department of Clinical Laboratory, School of Medicine, Pontificia Universidad Católica de Chile, 7820436 Santiago, Chile; 3 Biomedical Research Center, School of Medicine, Universidad de Valparaíso, 2540064 Valparaíso, Chile; 4 Cellular Signaling and Differentiation Laboratory (CSDL), School of Medical Technology, Health Sciences Faculty, Universidad San Sebastian, 7510156 Santiago, Chile; 5 Department of Hydraulic and Environmental Engineering, School of Engineering, Pontificia Universidad Católica de Chile, 7820436 Santiago, Chile; 6 Cellular Signaling and Differentiation Laboratory, School of Medical Technology, Medicine and Science Faculty, Universidad San Sebastian, 7510156 Santiago, Chile

**Keywords:** Biofilms, Medical research

## Abstract

Chronic wounds cannot heal due to impairment of regeneration, mainly caused by the persistent infection of multispecies biofilms. Still, the effects of biofilm wound infection and its interaction with the host are not fully described. We aimed to study functional biofilms in physiological conditions in vitro*,* and their potential effects in health and regeneration in vivo*.* Therefore, *Pseudomonas aeruginosa, Staphylococcus aureus* and *Enterococcus faecalis* were seeded in collagen-based scaffolds for dermal regeneration. After 24 h, scaffolds had bacterial loads depending on the initial inoculum, containing viable biofilms with antibiotic tolerance. Afterwards, scaffolds were implanted onto full skin wounds in mice, together with daily supervision and antibiotic treatment. Although all mice survived their health was affected, displaying fever and weight loss. After ten days, histomorphology of scaffolds showed high heterogeneity in samples and within groups. Wounds were strongly, mildly, or not infected according to colony forming units, and *P. aeruginosa* had higher identification frequency. Biofilm infection induced leucocyte infiltration and elevated interferon-γ and interleukin-10 in scaffolds, increase of size and weight of spleen and high systemic pro-calcitonin concentrations. This functional and implantable 3D biofilm model allows to study host response during infection, providing a useful tool for infected wounds therapy development.

## Introduction

Wound healing is a timely organized process that begin with haemostasis, followed by an inflammatory phase (1–3 days), a proliferative phase (4–21 days) and remodeling of the tissue extra-cellular matrix (ECM) that leads to wound contraction (21 days–1 year)^1^. When these highly regulated processes fail to progress, wounds will not be resolved becoming chronic.

Chronic wounds are a non-solved medical issue, with a rough prevalence rate of 1–2% among general population in developed countries^2^, mainly affecting the elderly, diabetic, obese, bedridden, surgical patients, and people with venous insufficiency^3^ or heavy smoking habits^4^. On average, chronic wounds can last for 12–13 months and most patients will recur despite treatment, severely affecting their life quality^5^. Around 26% of these wounds never heal, ending up with minor amputations^6^. In fact, globally, 85% of amputations are preceded by a chronic wound^7^ and, within 5 years, 70% of amputee diabetic patients died^8^. In addition to affect patient’s life quality, chronic wounds also comprise a major economic burden, as only treatment expenses are estimated to account for 1–3% of the total healthcare budget in developed countries^9^. Approximately 60% of chronic wounds are caused by the persistent infection of established multispecies biofilms that impair the regeneration process^10^. Several bacterial species can colonize the wound bed, forming viable biofilms, a lifestyle in which they are embedded in a self-produced polymeric matrix or “Extracellular Polymeric Substances” (EPS)^11^. This tridimensional structure provides bacterial biofilms an optimal environment to evade host immune responses and tolerate antibiotic treatment^12^. In the tissue, the persistent biofilm infection causes a prolonged chronic inflammation state, disrupting the normal healing process^12^. Moreover, biofilms provoke tissue hypoxia by consuming oxygen^13,14^, as well as different nutrients obtained from the exudate, thus depriving cells of the energy required for regeneration^15^. In addition, biofilms induce malfunction of epithelial tight junctions, leading to trans-epidermal water loss^16^, consequently losing the skin barrier function, allowing new bacterial cells to colonize and grow.

Biofilms infecting chronic wounds are usually multispecies, containing predominant pathogens such as *Pseudomona aeruginosa, Staphylococcus aureus* and *Enterococcus faecalis*, as well as commensal species such as *Corynebacterium spp.* and *Staphylococcus epidermidis*^17^. The established biofilms can exceed bacterial loads of 10^5^ colony forming units (CFU)/gram of wound tissue as determined by wound cultures of deep-tissue biopsies, needle aspiration, and swab cultures^18^, which has been proposed as a marker of infection in chronic wounds^10^, regardless of their microbial composition^19^. It has also been reported that 93% of all chronic wound infections are multispecies, whereas only 7% of those are single species, frequently dominated by *P. aeruginosa s*trains^17^. A prevalence of fastidious pathogenic strains has been detected in biofilms of chronic wounds from burnt patients, where Methicillin-resistant *S. aureus* (MRSA) and Vancomycin-resistant *Enterococcus* (VRE) strains display broad spectrum antibiotic tolerance^20,21^.

Because of its critical impact, in vitro and in vivo biofilm models have been developed to study their role in wound healing. Some of these models include biofilms formed on clinical wound care supplies like sutures^22^, absorbent pads^23^, gauzes^24^, silver coated^25^ or antimicrobial wound dressings^26^. Other models have used biological substrates for biofilm formation, such as plasma with horse red blood cells^27^, human plasma^28^, collagen coated slides^29^, collagen gel^30^, a scaffold composed of hyaluronic acid and collagen^31^, and skin explants for ex vivo models^32^. These models allowed characterization of biofilm phenotypes, cell attachment to materials and testing of novel antimicrobials, but most of them have not been implanted for subsequent in vivo analysis. Suture models of monospecies infection have been implanted in mice, but the use of monospecies models lack the polymicrobial nature of biofilms in actual wounds^33^.

Several in vivo models have also been established, mainly in mice and pigs, and most of them rely on the use of single-species inoculation of *Pseudomonas* spp. or *Staphylococcus spp.* in planktonic state that can form biofilms and induce tissue damage^34^, collagenolytic MMP-2 activity and collagen synthesis inhibition^35^, or even septicemia^36^, resulting in delayed wound healing. Other authors have used two-species combinations to infect wounds in mice for efficacy and safety testing of glycoside hydrolases^37^, in horses evaluating normal and impaired regeneration^38^, or in pigs causing disruption of the skin barrier function^16,27^. Regarding wound dynamics, in mouse models regeneration occurs mainly by contraction, instead of regeneration that drives healing in human wounds^38^. Regarding the infection process, although these models have provided valuable insights in terms of biofilm virulence, wound closure and bacterial loads, most of them have not characterized the systemic and local effects of biofilm infection on general health parameters of the host, and how such biofilms affect the wound healing process.

Taking the above-mentioned aspects in consideration, the aim in this work was to establish a reliable in vitro and in vivo model resembling the tridimensional complex interactions that are established between multispecies biofilm and the biological substrate, as well as the host and pathogens interactions, and their effect during the wound healing process.

## Results

### Formation of biofilms in 3D scaffolds in vitro

**Figure 1:**
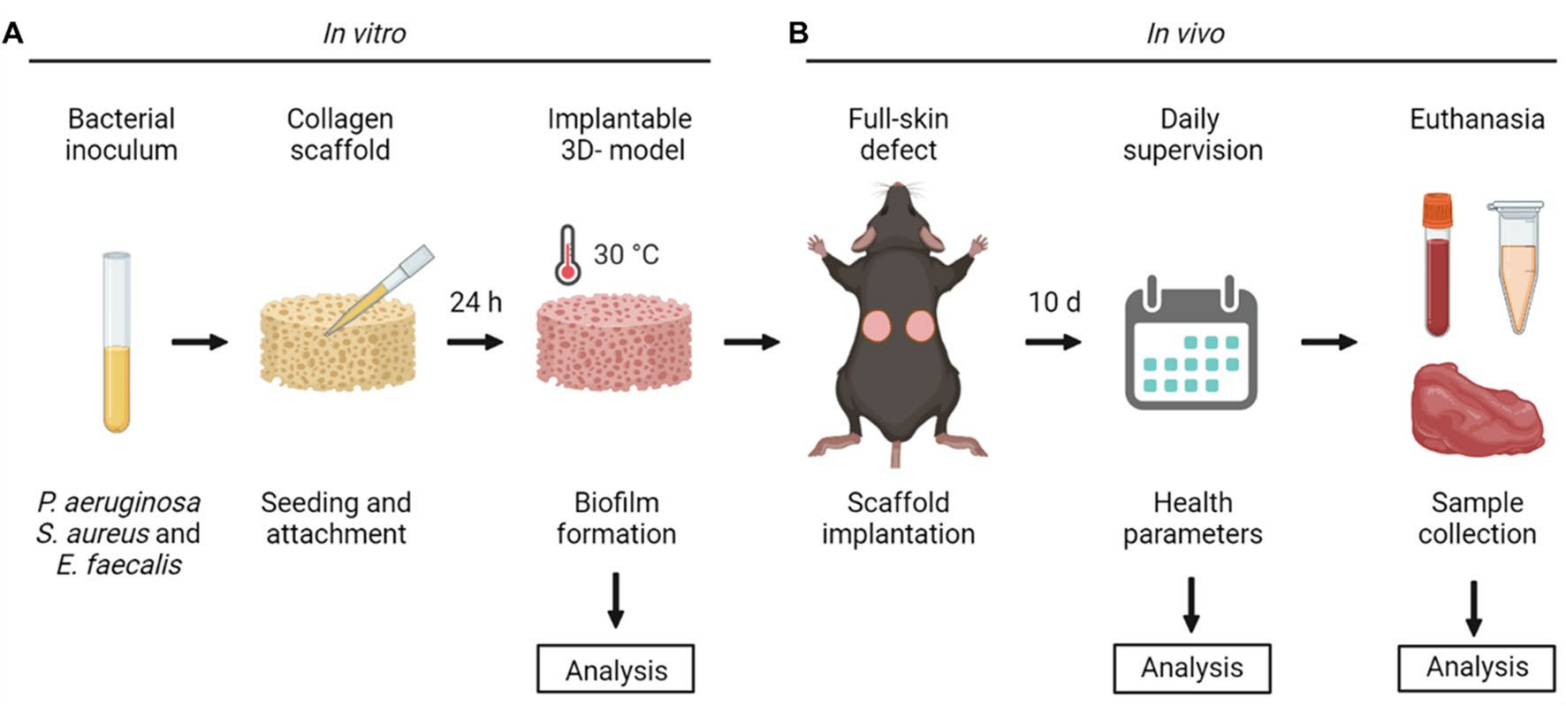
Schematic representation of the in vitro and in vivo biofilm model. (**A**) Strains from *P. aeruginosa*, *S. aureus* and *E. faecalis* were diluted and seeded on collagen scaffolds to induce biofilm formation. After 24 h of incubation, the biofilm in vitro model was analyzed in means of metabolic activity, bacterial loads, structure and antibiotic tolerance. (**B**) Once characterized, biofilm-containing scaffolds were implanted in bilateral full-skin defects in mice during 10 days to evaluate the effect over general health parameters through daily supervision. Further analysis of the animal samples were performed, including histology and immunohistochemistry, bacterial loads, pro-calcitonin and cytokine levels. Image was made with BioRender.

**Figure 2:**
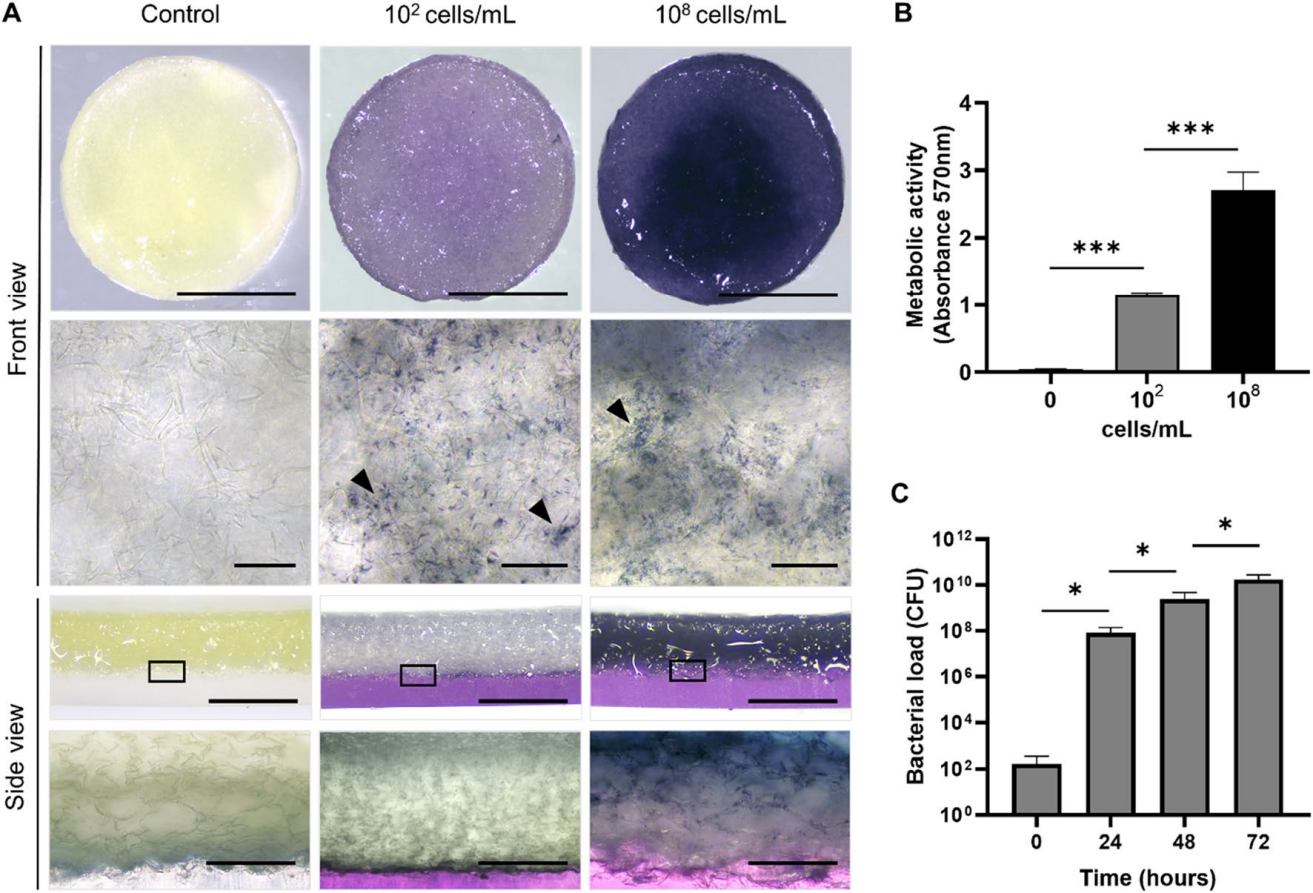
Viability, distribution, and bacterial loads of biofilms formed in scaffolds. (**A**) MTT assay of scaffolds seeded with 10^2^ or 10^8^ cells/mL or without cells (control) and incubated for 24 h showed equal distribution of viable biofilms across the scaffolds in both, front and side views. In higher magnification of scaffolds, bacterial accumulation was observed (arrow heads). (**B**) Quantitation shows that metabolic activity increased according to the initial cell density. (**C**) Biofilm-containing scaffolds were diluted and plated in TSS agar for CFU counting. Quantitation shows over 10^5^ CFU/scaffold after 18 h incubation of scaffolds, and up to 10^10^ CFU/scaffold after 72 h of bacterial growth. Scale bar for A represents 10 mm (front view), 50 µm (upper zoom), 0.5 mm (side view), 240 µm (lower zoom). Values plotted are mean ± SD (N = 3). One-way ANOVA and Tukey’s posterior comparison for B and unpaired t-test, comparing each time point with their previous condition for C. *p < 0.05; ***p < 0.001.

As described in the material and method section, a mixture of *P. aeruginosa*, *S. aureus* and *E. faecalis* was incorporated in a 3D collagen-based scaffold that is clinically used for dermal regeneration (Fig. 1). After 24 h of incubation, bacteria remained metabolically active as shown by formation of formazan blue in the MTT (1-(4,5-Dimethylthiazol-2-yl)-3,5-diphenylformazan) assay. Macroscopic visualization of scaffold’s front view (Fig. 2A, upper panel) showed homogeneous distribution of bacteria across the samples seeded with low bacterial loads, whereas in samples with higher loads an increase in the formation of formazan blue was observed in the center of the scaffolds. At both low- and high-density loads, higher magnification showed the presence of bacteria attached to the scaffold fibers, forming a tridimensional structure. Side view images shows a homogeneous vertical distribution at lower bacterial loads, with bacterial aggregates evenly distributed across the scaffolds, while crystals were predominantly concentrated in the upper region of the scaffolds when bacteria were seeded at higher loads (Fig. 2A, lower panel).

No MTT reduction was observed in control non-seeded scaffolds, whereas a quantitative analysis showed a significant difference among the groups seeded with high and low bacterial loads, which was not directly proportional to the seeded bacterial number (Fig. 2B). CFU counts per scaffold were 10^2^ CFU/mL at day 0 (Fig. 2C) and increased to 10^6^ CFU/mL in 24 h, reaching 10^10^ CFU/mL in 48 h (Fig. 2B).

**Figure 3:**
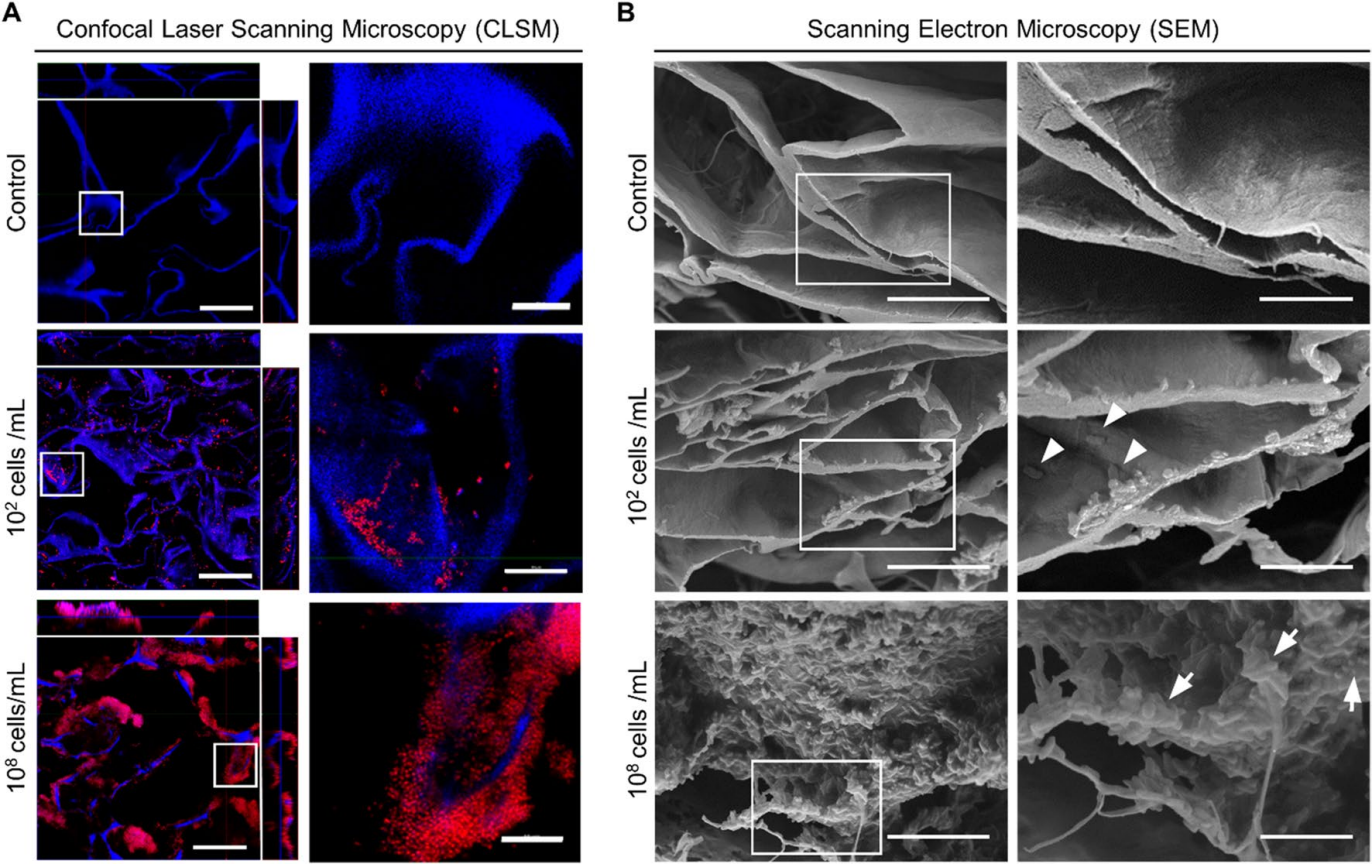
Microscopy characterization of biofilms formed in the scaffolds. (**A**) Fixed scaffolds were stained with PI (red) and DAPI (blue) for CLSM visualization, showing biofilm formation on scaffold fibers and bacterial aggregates (zoomed right panels). Side images from left panels represent a Z-view of two optical sections (X- and Y-view) from the scaffold. (**B**) SEM analysis shows individual cells (arrowheads) and bacterial aggregates (arrows) with EPS formation (zoomed right panels). Scale bars for A represent 50 µm (left panel) and 10 µm (right panel); for B represent 12 µm (left panel), 5 µm (right panel). In A, blue signal is present in all groups due to collagen autofluorescence.

To visualize the structure of seeded microorganisms over the biomaterial, scaffolds were analyzed by different microscopic techniques. Confocal laser scanning microscopy (CLSM) analysis showed bacterial aggregates attached to the surface of the scaffold, forming a biofilm-like structure, which was more prominent in the scaffolds seeded with higher bacterial loads (Fig. 3A, upper panels). Z-view shows that bacteria distributed homogeneously in the surface, with the presence of bacterial colonies established in the inner cavities of the material (Fig. 3A, lower panels). Scanning electron microscopy (SEM) revealed a more detailed analysis of the biofilm structure, showing a rather smooth surface in the control non-seeded scaffolds, compared to scaffolds containing bacteria that fully colonize the surface of the material at higher bacterial loads (Fig. 3B). Additionally, SEM images revealed that most bacteria spotted in biofilm-containing scaffolds are rod-shaped cells embedded in noticeable amounts of extra polymeric substance (EPS).

### In vitro functionality of biofilms formed in 3D scaffolds

**Figure 4:**
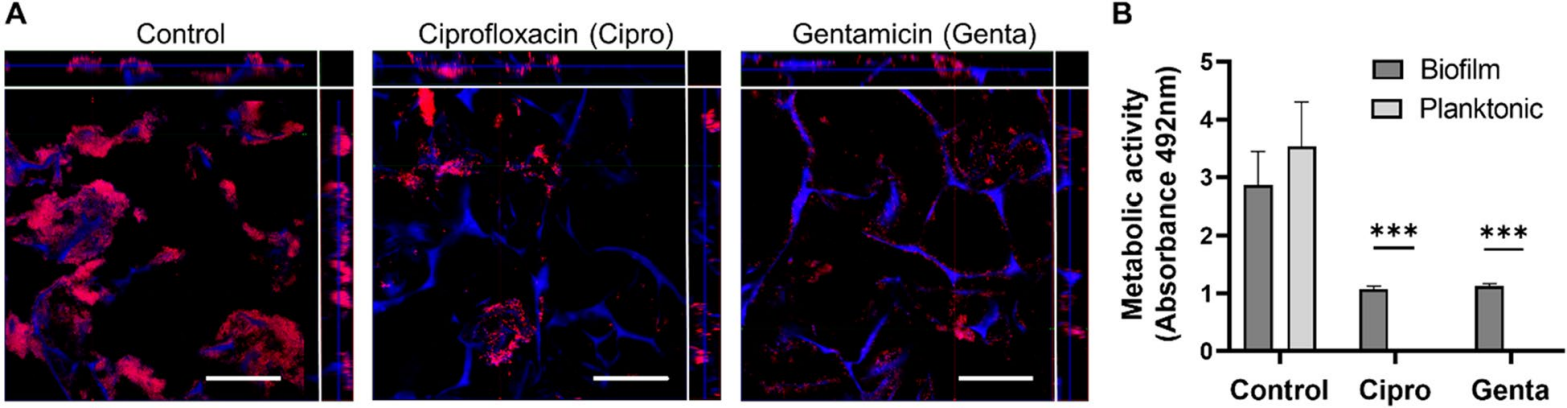
Broad-spectrum antibiotics treatment of biofilms formed in the scaffolds. (**A**) Biofilm-containing scaffolds treated with antibiotics were fixed and stained with PI (red) and DAPI (blue) for confocal microscopy visualization, showing remaining biofilms. Side images from left panels represent a Z-view of two optical sections (X- and Y-view) from the scaffold. (**B**) Viability assay (XTT) of biofilms formed over scaffolds and treated with antibiotic confirms a significantly higher metabolic activity compared to planktonic cultures. Scale bar represent 50 µm in A. Values plotted are mean ± SD (N = 3). Two-way ANOVA and Sidak’s multiple comparisons test. ***p < 0.0001. In A, blue signal is present in all groups due to collagen autofluorescence.

Once the presence of viable biofilms in 3D scaffolds was confirmed, we decided to study their functionality in terms of antibiotic tolerance, by comparing them to planktonic cultures of the same species. Biofilm-containing scaffolds and bacterial planktonic suspensions were treated with high concentrations of ciprofloxacin or gentamicin, and their antibiotic effect was analyzed (Fig. 4). The microscopic visualization of scaffolds shows a clear reduction of bacterial biofilm biomass after treatment with each antibiotic, where bacteria directly attached to the scaffold preserved a biofilm-like structure (Fig. 4A), remaining attached to cavities within pores of the biomaterial, regardless of the antibiotic type. A minimum inhibitory concentration (MIC) assay of planktonic cultures (Supplementary Fig. S1) showed that a concentration of 10 µg/mL of gentamicin and 1 µg/mL of ciprofloxacin inhibited planktonic bacterial growth and activity, which was confirmed through measurement of OD 600 nm and XTT (2,3-Bis(2-methoxy-4-nitro-5-sulfophenyl)-2H-tetrazolium-5-carboxanilide) reduction assays. To quantify this effect, an XTT assay of planktonic cultures and biofilm-containing scaffolds treated with antibiotics (Fig. 4B) showed no metabolic activity for planktonic cells, while biofilms remained viable after treatment with each antibiotic. No statistical differences were found between planktonic and biofilm cells in control media.

### Implantation of biofilm-containing scaffolds in vivo

**Figure 5:**
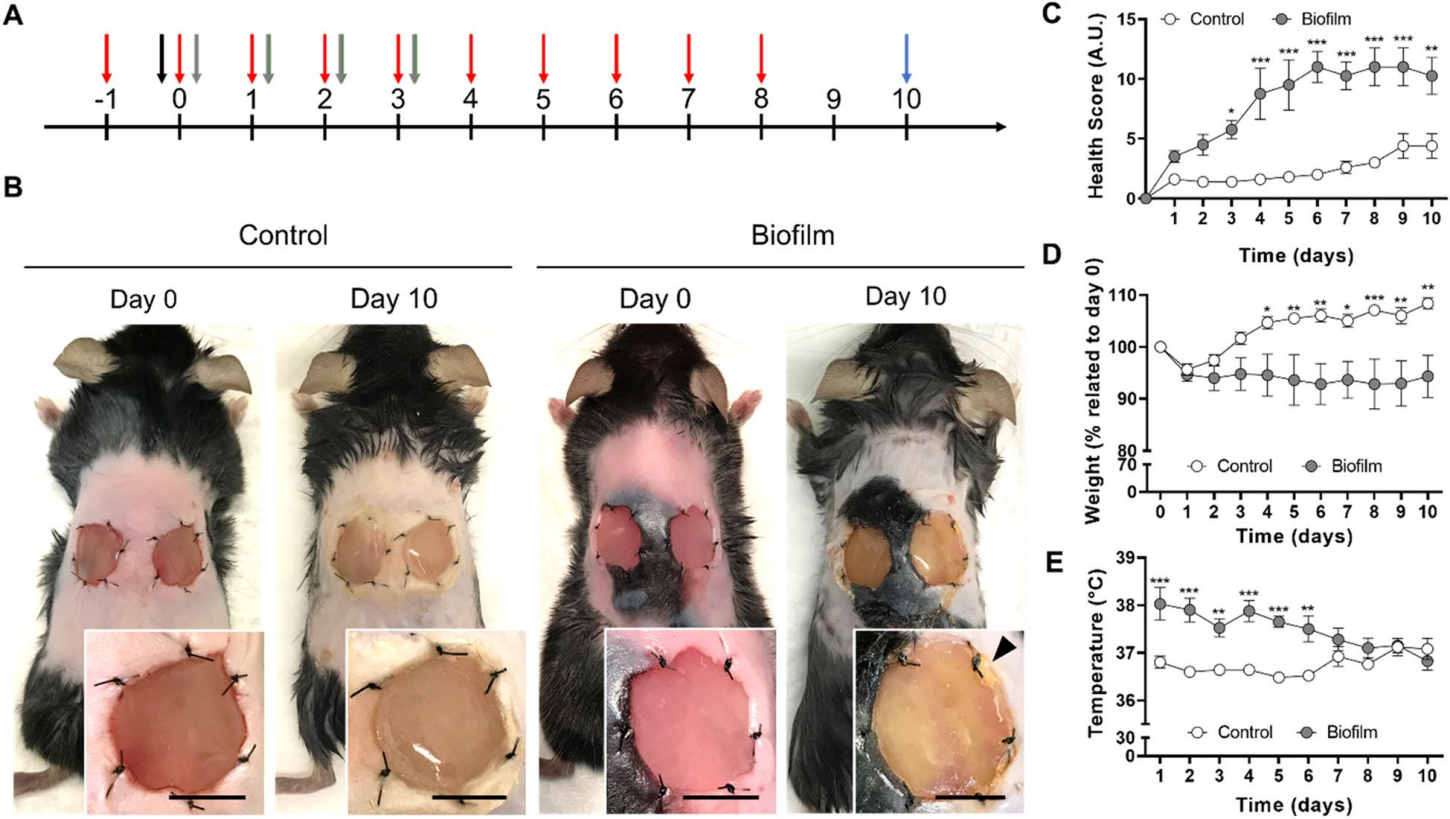
Time course of mice welfare during 10 days of biofilm implantation. (**A**) Timeline of the in vivo model, showing treatments and supervision timing: ciprofloxacin IP 30 mg/kg (red), surgery (black), meloxicam SC 5 mg/kg (gray), euthanasia (blue). All animals were daily supervised according to ethical protocols for general health assessment. (**B**) Representative images of control or biofilm-infected wounds in the scaffold dermal regeneration model immediately after surgery (Day 0), and after 10 days of implantation (Day 10). Arrow head indicates secretion surrounding the wound area. (**C**) Health score for assessment of welfare, indicating that biofilm-infection affects animal’s health from Day 3 to Day 10. (**D**) Relative body weight (% of initial weights at day 0), shows that biofilm-infected group did not recover their weight. (**E**) Body temperature shows that biofilm-infected group suffers from initial fever but stabilizes temperature at latest days. Scale bar represents 10 mm for B. Values plotted are mean ± SD (N = 8 per group). Two-way ANOVA and Sidak’s multiple comparison test. *p < 0.05; **p < 0.01; ***p < 0.001.

After characterization of the in vitro 3D biofilm model, an in vivo study was performed. In a pilot experiment, biofilm-containing scaffolds were implanted on bilateral full-thickness skin wounds in mice. Despite adequate analgesia, animal care and cauterization of bleeding vessels during surgery, all mice died from sepsis after one-two days of implantation (Supplementary Fig. S2). To prevent this, further in vivo assays included the use of antibiotic and antipyretic treatment. Ciprofloxacin and meloxicam were daily administered to animals (Fig. 5A) resulting in 100% mice survival after biofilm-containing scaffold implantation. During the first two days a few mice showed bleeding around the suture knots, and after ten days there were no signs of bleeding around the scaffolds nor significant wound contraction in either group (Fig. 5B). Implanted sterile scaffolds (control), showed no macroscopic signs of infection or inflammation. In contrast, some animals with biofilm-containing scaffolds showed a yellow secretion in the surrounding wound areas or under the scaffold (Fig. 5B).

A daily supervision of animal’s health showed a detrimental effect of biofilm implantation over global health parameters (Fig. 5C). Thus, the health score obtained from the daily supervision guide, was significantly increased since day 3 in mice implanted with biofilm-containing scaffolds. A significant difference in body weight was also observed between both groups, as biofilm-implanted mice did not recover their initial weight (Fig. 5D). Finally, during the first four days, the biofilm-implanted group showed a significant increase in the body temperature at the back of mice, which was recovered after five days (Fig. 5E).

### Systemic and local infection process due to biofilm implantation

**Figure 6:**
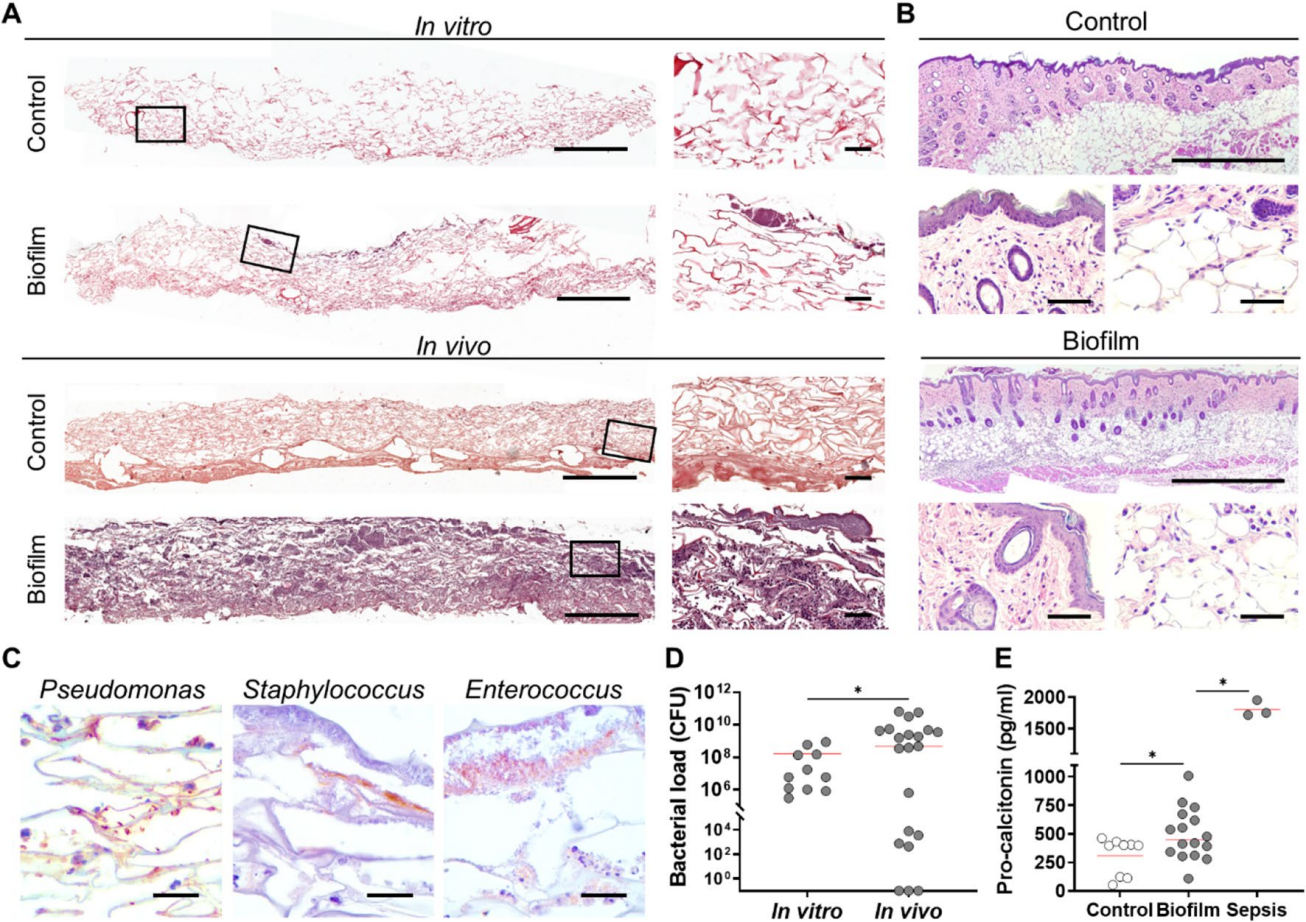
Infection of wounds after implantation of biofilm-containing scaffolds. (**A**) Histological sections from scaffolds in vitro (pre-implantation), and in vivo (10 days of post-implantation) were stained with H&E. (**B**) Histological sections from skin samples of the wound edge were stained with H&E, showing infiltration of leucocytes in the adipose tissue (right lower panels) but not in the epidermal layer (left lower panels). (**C**) Histological immune staining, showing bacterial aggregates corresponding to each bacterial species. (**D**) Scaffold samples were diluted and plated in TSS agar for CFU determination, showing over 10^6^ CFU/gr in vitro (N = 11) and 10^9^ CFU/gr for the in vivo group (N = 21). (**F**) Pro-calcitonin levels were measured from plasma of animals implanted with sterile scaffolds (control), biofilm-containing scaffolds with antibiotics (Biofilm), and biofilm-containing scaffolds without antibiotics provoking septicemia (Sepsis). Scale bar for A represents 1000 µm (left) and 60 µm (right); for B represents 1000 µm (upper) and 60 µm (lower); for C represents 20 µm. Individual values and mean (red line) are plotted. Unpaired t-test, comparing with corresponding previous group. *p < 0.05.

Next, scaffolds were removed, and the effect of a possible infection process was studied. Here, paraffin sections of biofilm-containing scaffolds prior to implantation and after ten days in vivo were sectioned and stained for histological analysis (Fig. 6A). Bacterial aggregates were mainly accumulated in the upper region of the biomaterial before implantation, but after ten days such bacterial aggregates were widespread over the scaffold. Sterile implanted scaffolds show cellularization of fibroblast, without leucocyte infiltration, compared to biofilm-implanted scaffolds that showed a considerable amount of polymorphonuclear (PMN) cells, embedded within an abundant biofilm structure. Hematoxylin and eosin (H&E) stained sections of skin surrounding wound area (Fig. 6B) did not show considerable differences due the presence of bacteria, except for an increased presence of leukocyte infiltrate in the adipose tissue of the biofilm model.

To discard a systemic infection or sample cross contamination, blood collected from animals was analyzed for CFU counting. Results showed that in both groups no bacterial growth was observed in blood agar plates (Data not shown). To corroborate the presence and distribution of bacterial species seeded in the scaffold, these were identified in situ using specific antibodies (Fig. 6C). Then, CFU/g of scaffolds were quantified (Fig. 6D), comparing scaffolds before implantation (in vitro) and after ten days in vivo. Control scaffolds showed to be sterile, with no bacterial growth, whereas biofilm containing scaffolds in vitro showed mean values of 10^7^ CFU/g. Once implanted in full thickness wounds, biofilms reached over 10^9^ CFU/g of scaffold despite the ciprofloxacin therapy. To further evaluate the infection process induced by biofilms, pro-calcitonin levels were quantified in serum (Fig. 6E). Although pro-calcitonin basal levels were high for animals implanted with sterile scaffolds (control), biofilm implantation together with antibiotic therapy resulted in significantly increased levels, with over 500 pg/mL of pro-calcitonin (biofilm), while animals that suffered septicemia (N = 3) caused by biofilm implantation without antibiotics (sepsis) reached levels over 1.500 pg/mL.

To identify bacterial species in the scaffolds, MALDI-TOF MS (Matrix-assisted laser desorption/ionization time of flight mass spectrometry) analysis was performed (Supplementary Fig. S3), showing the predominance of *P. aeruginosa* in 90% of samples before and after 10 days of implantation, 10% of samples had *E. faecalis* and 10% had *S. aureus*. Also, several other bacterial genera that were not initially inoculated were found in colonized wounds, but they were specific for each specific wound, where 20% of samples had *Kocuria rosea*, 10% had *Escherichia coli*, 10% had *Pseudarthrobacter sulfonivorans* and 10% had *Pseudomonas straminea*, showing a total of seven bacterial species detected in biofilm-containing scaffolds from this study (data not shown).

### Systemic and local inflammation process induced by local infection

**Figure 7:**
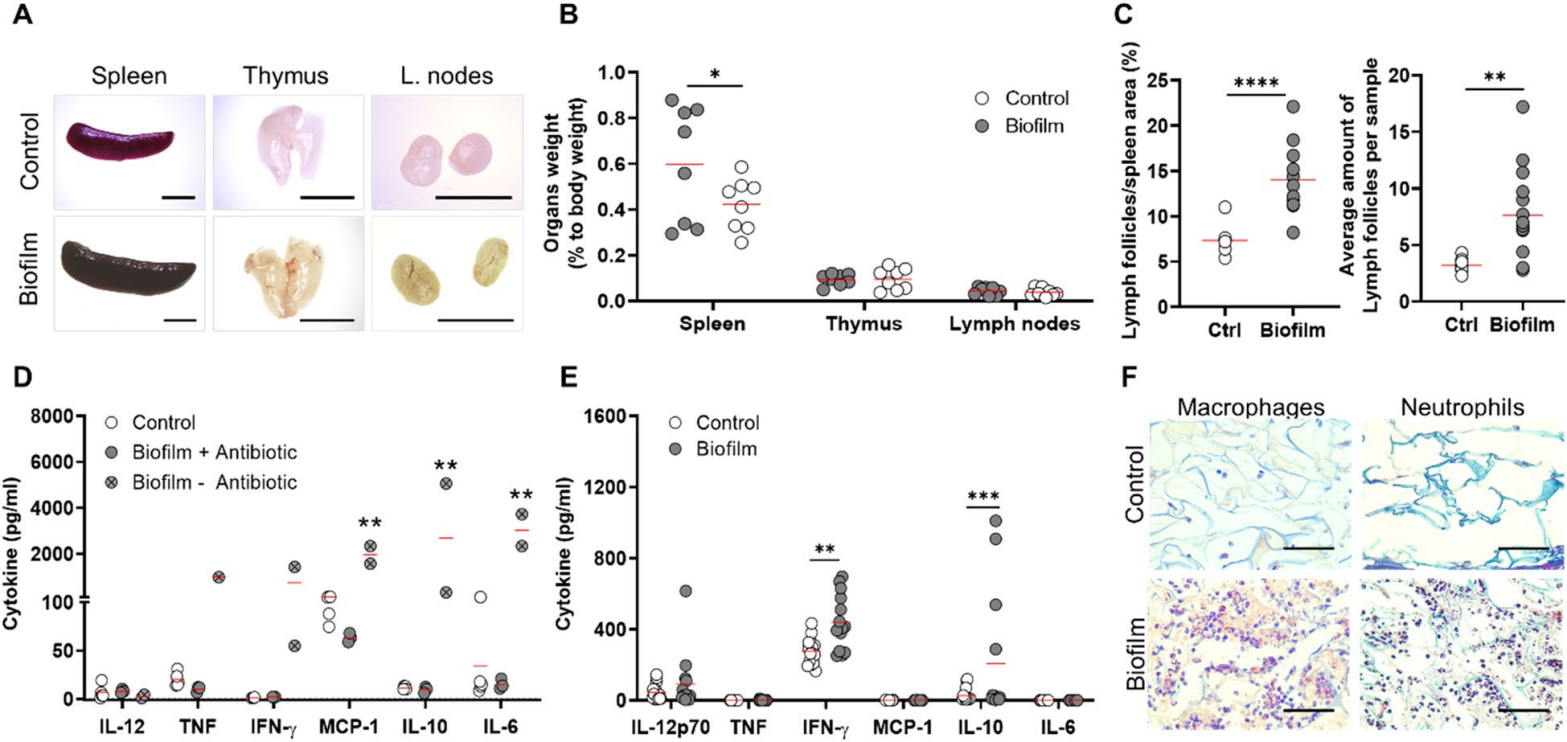
Systemic and local inflammatory response of mice towards biofilm-containing scaffolds. (**A**) Representative images of immune organs show increased size of spleen due to biofilm implantation. (**B**) Organs weight relative to total body weight of mice show a significant increase of spleen due to biofilm infection, whereas thymus and lymph nodes show no differences. (**C**) Morphometrical analysis of spleen slices stained with H&E to quantify are of lymph follicles per spleen area and average number of lymph follicles per sample. (**D**) Plasma samples from mice implanted with sterile scaffolds (Control), with biofilm containing scaffolds and ciprofloxacin systemic treatment (Biofilm + antibiotic) or no antibiotics (Biofilm – antibiotic) were analyzed for pro-inflammatory cytokine quantitation. Only animals with infected scaffolds and no systemic treatment had significantly higher cytokine levels. (**E**) Protein extracts from the implanted scaffolds were analyzed, showing higher levels of IFN-γ and IL-10 due to biofilm infection. (**F**) Histological sections from biofilm-containing or control scaffolds after implantation were processed for immunohistochemistry with CD68 antibody (macrophages) and Masson’s trichrome stain (neutrophils). Representative images show infiltration of macrophages and PMN cells in presence of biofilm. Scale bar for A represents 5 mm; for F represents 60 µm. Individual values and mean (red line) are plotted. Two-way ANOVA and Sidak’s multiple comparison test for B and E; Mann–Whitney test for C; and two-way ANOVA and Tukey’s posteriori comparison for D. *p < 0.05; **p < 0.01; ****p < 0.0001.

After confirming a local infection response provoked by the biofilm implantation in wounds, their effect over the systemic inflammation process was characterized (Fig. 7). For systemic inflammation analysis, blood and lymphoid organs were extracted. Increased size of the spleen was observed due to biofilm implantation (Fig. 7A), which was confirmed with a significant increase in its weight, compared to the control group (Fig. 7B). Lymph nodes and thymus did not show variations in size nor weight under biofilm infection (Fig. 7A,B). The microscopic inspection of spleen histological slides stained with H&E showed an increase in the ratio of lymph follicles/spleen area and the average amount of lymph follicles in the biofilm-infected groups (Fig. 7C).

To quantify systemic pro-inflammatory cytokines, blood was extracted from implanted animals, and plasma fraction was analyzed by flow cytometry (Fig. 7D). None of the cytokines measured showed significant variations between groups treated with ciprofloxacin (control or biofilm-infected groups). In contrast, cytokine levels from animals with biofilm-containing scaffolds that were not treated with antibiotics, and suffered septicemia, showed elevated levels for cytokines MCP-1 (monocyte chemoattractant protein-1), IL-10 and IL-6 compared to groups treated with ciprofloxacin and control (Suppl. Fig. S2).

Once the systemic inflammatory response was evaluated, the local inflammatory process was also studied (Fig. 7E,F). Here cytokines obtained from protein extracts of the implanted scaffolds were quantified, showing significantly higher levels of IFN-γ and IL-10 in infected samples compared to sterile ones (Fig. 7E). To evaluate leukocyte infiltration, histological sections from scaffolds were processed for immunohistochemical and histochemical analysis to detect macrophages and neutrophils (Fig. 7F). Results show that in the presence of biofilm there was a marked response from the host, given by the higher presence of macrophages and neutrophils in the scaffold.

**Figure 8:**
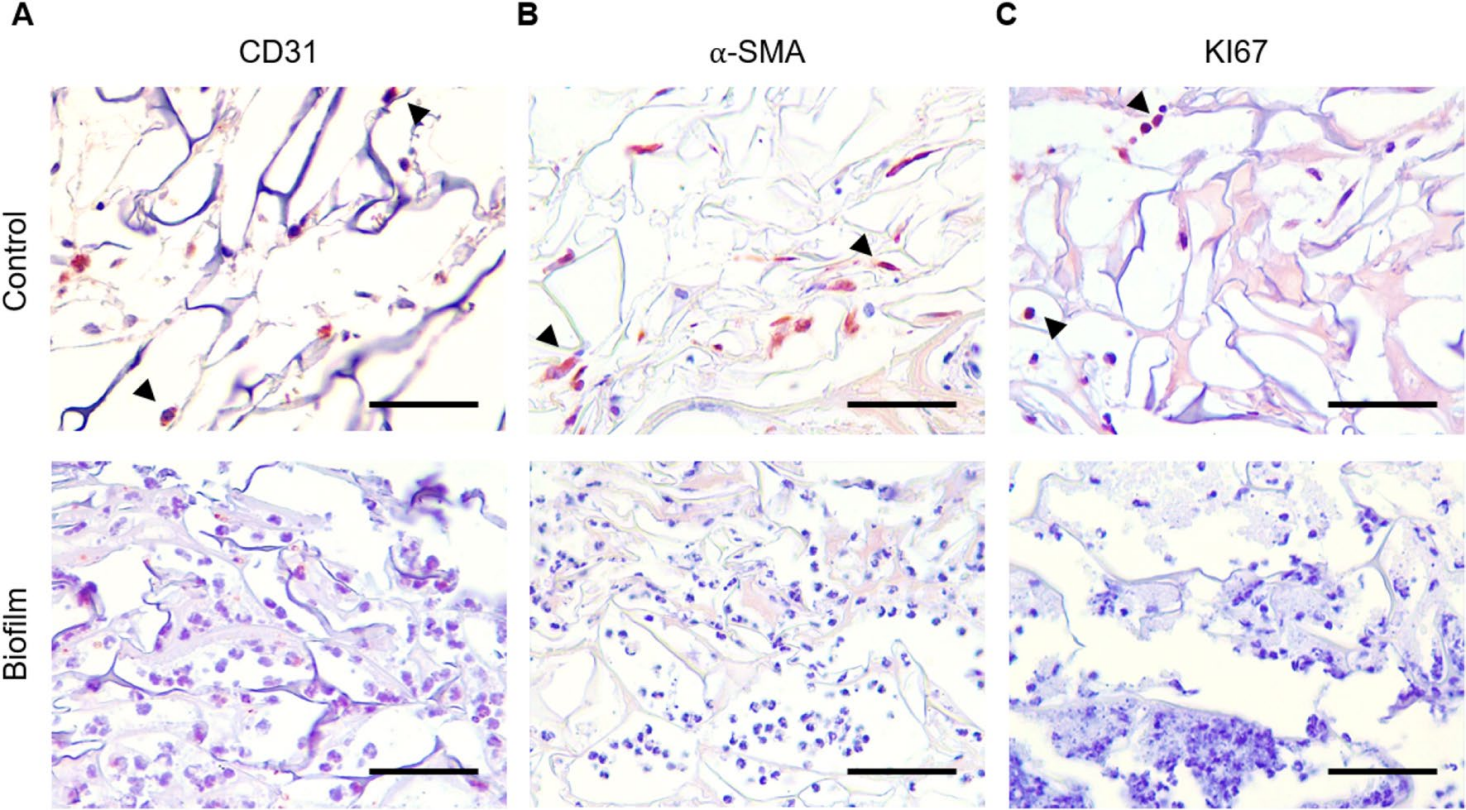
Regeneration parameters in vivo. Histological sections from biofilm-containing or control scaffolds after ten days of implantation were processed for immunohistochemistry with specific antibodies and a qualitative assay was performed. (**A**) Endothelial cells as an indicator of angiogenesis analyzed with CD31. (**B**) Activation of fibroblasts in the wound site analyzed with α-SMA antibody. (**C**) Cell proliferation analyzed with KI67 antibody. Positive stained cells are indicated with arrow heads. Scale bar represents 60 µm.

To qualitatively assess the effect of biofilm in the regeneration process, paraffin sections were processed for Masson’s trichrome stain and immunohistochemistry (Fig. 8). Results showed the presence of endothelial cells marked with cluster of differentiation 31 (CD31, Fig. 8A), myofibroblasts with alpha-smooth muscle actin (α-SMA, Fig. 8B) and cells under cell proliferation (ki67, Fig. 8C), consistent with the regeneration process in control animals implanted with sterile scaffolds. In the case of biofilm-infected groups, these markers were not visualized, while a high number of leucocyte infiltrate was observed.

## Discussion

Despite decades of research, the role of biofilm-mediated infection in chronic wounds has not yet been completely elucidated, hence the development of in vitro and in vivo biofilm models to better understand their biology, as well as their role in the regeneration process, is required to develop novel strategies for chronic wound management. Thus, the aim of this work was to establish a biofilm-infected in vitro model, which can be further implanted in a wound model to evaluate the effect of biofilms in vivo*.*

Initially, conditions were established to grow biofilms in commercially available collagen-GAG based scaffolds, that resemble the skin extracellular matrix in terms of structure and composition, which has been broadly described in research and for its clinical use for dermal regeneration^39^. Using an MTT assay, where formazan crystals remain intracellularly^40^, viable bacteria within the biomaterial were localized and quantified^40^. Results confirmed the formation of a metabolically active biofilm formed on the biomaterial’s surface, which, for low cell density biofilms, was homogeneously distributed, while those formed from high bacterial loads were at the center and bottom of the scaffold. This suggests the formation of metabolic gradients due to oxygen or nutrient’s chemical gradients^11^, which can correlate with their ability to tolerate antibiotics^41^. CLSM and SEM analysis was consistent, demonstrating that biofilms organized in bacterial aggregates attached to the pores of the scaffold. Possibly, biofilm formation started with bacteria that attach to the surface and latter multiply, secreting EPS to become a thick-layer biofilm established in the scaffold, following a typical growth cycle^42,43^.

In the present in vitro model, tolerance against ciprofloxacin and gentamicin treatment was corroborated in biofilms formed over collagen-scaffolds, as previously reported for *P. aeruginosa* over gels (with 10^2^ CFU/gel after 24 h)^30^ and mixed-species biofilms in the Lubbock model that reaches approximately 10^5^ to 10^6^ CFU/gr of tissue after 5 h^44^. Our results showed a 100- and 20-fold increase in the MIC of ciprofloxacin and gentamicin for bacteria forming biofilms compared to planktonic, as described for chronic wound biofilms^45,46^, indicating that the proposed in vitro model is viable and functional. Moreover, in vivo results confirmed that ciprofloxacin therapy prevents septicemia, since bacterial inoculation without treatment resulted in 100% mortality of mice, an outcome previously reported in mice and horse models^36,38,47^. Despite the daily dose of ciprofloxacin 10 mg/kg, biofilm implantation generated a persistent infection, with a detrimental effect over animal welfare as seen in higher health score, permanent weight loss and initial febrile state, resembling the severely affected life quality of chronic wound patients^5^.

The model described here demonstrates that a low-density initial inoculum can form biofilms of 10^8^ CFU/mL after 24 h, and reach over 10^9^ CFU/gr of tissue after ten days in vivo, resembling a more clinically relevant initial bacterial exposure to patients^48^. Similarly, CFU counting revealed groups of non-infected, mildly, or highly infected wounds, which correlated with gradients of infiltration and migration of PMN cells and macrophages visualized by histology. This observation mimics the categorization of patient’s wounds into ‘light’, ‘occasional’, ‘moderate’, or ‘heavy growth’ infected^49^. Regarding the bacterial diversity^50^ despite *Pseudomonas* dominated the infection process in most animals, there was a less frequent presence of *S. aureus*, *E. faecalis*, and the occasional identification of seven bacterial species from mice and the environment, which suggests that the proposed scaffold-infected model has a heterogeneous polymicrobial and dynamic composition like chronic wounds^17^.

At a systemic level, an increased size of spleen and histological analysis shows that implantation of biofilm-containing scaffolds induce splenomegaly, with increased size and number of lymph follicles where lymphocyte T and B differentiate and proliferate^52^, suggesting a high immunological activity of the spleen probably in response to bacterial lipopolysaccharides, as it has been previously reported^53^. In addition, levels of pro-calcitonin were significantly higher for biofilm-infected groups compared to controls, with protein concentration values in plasma within reported ranges for chronic-infected patients^54,55^, demonstrating that, besides detrimental effect over welfare, other systemic effects that mimics patient’s symptoms were also present.

Locally, quantitation of cytokines from the implanted scaffolds showed an increase in IFN-γ and IL-10 cytokines, while pro-inflammatory cytokines IL-12, TNF-α, IL-6 and MCP-1 did not change. Interestingly, it has been reported that IL-10 can be enhanced by pathogens^56–58^ through TLR activation in macrophages by *S. aureus*^59^, and P. *aeruginosa*^60^, inhibiting the production of IFN-γ, IL-12 and TNF-α^59^. Hence, biofilm-containing scaffolds may be inducing IL-10 as an evasion mechanism, perpetuating the local infective process. Regarding the morphology at the wound site, histologic analysis indicated two types of cellular interactions: at the wound edge, there was an influx of PMN cells as an inflammatory response against biofilm, whereas in the wound bed the low count of cells indicated inhibition of cellularization and epithelialization. Consistent with this observation, a qualitative histological analysis shows the lack of myofibroblasts, cells in a proliferative state and endothelial cells are linked to a reduced capacity for regeneration, suggesting that biofilm infection seriously impairs wound healing, as previously described in other animal models^34,35,38^.

In conclusion, this study provides a biofilm-infected wound model that resembles most clinical aspects of chronic wounds. The establishment of biofilms over a scaffold provides an implantable in vitro model, with tested antimicrobial tolerance and bacterial loads, which can be used for screening of therapies prior to implantation. In parallel, the in vivo wound model described here avoids wound contraction, resembling scaffold-based dermal regeneration in humans, reporting systemic and local effects of a polymicrobial biofilm infection over health and inflammation, similar to chronic wound patients’ symptoms. The incorporation of guidelines regarding the welfare and health state of animals and pro-calcitonin quantitation, which have not been assessed in other animal models, represents another contribution of this in vitro and in vivo infected wound model, making it suitable for testing novel therapies for chronic wound management and treatment.

## Materials and methods

### Biofilm formation in scaffolds

Bacterial strains of *P. aeruginosa* (ATCC 27,853), *S. aureus* (ATCC 29,213) and *E. faecalis* (ATCC 29,212) were cultured overnight at 30 °C in Luria Bertani broth, diluted (1:3) and incubated 1.5–2 h until exponential growth. Then, cells were harvested, counted, and resuspended to equal species proportions to 10^2^ or 10^8^ cells/mL, and seeded in 12-mm diameter and 2-mm thickness collagen-glycosaminoglycan scaffolds (Integra^®^ DRT, kindly provided by Integra LifeSciences), which were previously dried with a sterile gauze. After 30 min of initial bacterial attachment, scaffolds were dried again, rehydrated with 1 mL M9 minimal culture media and incubated at 30 °C for 24 h under static conditions to allow biofilm formation. To avoid retention of planktonic cells, supernatants were removed and biofilm-containing scaffolds were dried with sterile gauzes and processed for further analysis or animal implantation.

### MTT metabolic assays

Seeded or sterile scaffolds were incubated in 90 µL of M9 media containing 10 µL of 5 mg/mL MTT (Sigma Aldrich) for 4 h at 37 °C. Next, scaffolds were imaged using a stereomicroscope (Leica Biosystems). Afterwards, scaffolds were incubated in 500 mL dimethyl sulfoxide (Sigma Aldrich) until all formazan blue was dissolved. Absorbance of formazan blue was measured at 570 nm and 550 nm was used as reference^61^.

### Bacterial quantitation and species identification

For in vitro studies (“Biofilm formation in scaffolds” and “Antibiotic tolerance assay”), biofilm-containing scaffolds were dried with sterile gauzes and mechanically disrupted by pipetting in 0.5 mL PBS, 2 min vortex, serially diluted and seeded in Trypcase Soy agar + 5% sheep blood plates (Biomérieux). After 24 h of incubation at 30 °C, colony forming units (CFU) were quantified. For in vivo samples (Sect.  2.7), a quarter of each implanted scaffold was weighed, resuspended in 0.5 mL phosphate buffered saline (PBS) vortexed for 2 min, serially diluted and seeded for further CFU counting. For species determination, isolated colonies were identified by MALDI-TOF Mass Spectrometry (Bruker Daltonik) as previously published^62^. The colonies were identified according to the database provided by the manufacturer: MALDI Biotyper library v4.0 5.627 MSP using the MALDI Biotyper 3.1 software package (Bruker Daltonik), using default settings. Identification scores of ≥ 2.0 indicated species‐level identification.

### Confocal laser scanning microcopy and image processing

Scaffolds from in vitro studies (“Biofilm formation in scaffolds” and “Antibiotic tolerance assay”), were dried using sterile gauzes, fixed with 0.5 mL of 4% paraformaldehyde in PBS for 1 h at room temperature, dried with gauze and washed three times with distilled water. For bacterial visualization scaffolds were stained for 10 min with 25 µL of propidium iodide (25 µM), washed three times with water and stained with 25 µL DAPI 5 µM in 0.01% formaldehyde for 10 min, washed once again with water and mounted for visualization by CLSM (Zeiss LSM 710 confocal microscope system). Images were processed with the ZEN Zeiss 3.4 (blue edition) software^63^.

### Scanning electron microscopy (SEM)

Biofilm-containing scaffolds, seeded with 10^2^ or 10^8^ bacteria/mL and incubated for 24 h at 30 °C (“Biofilm formation in scaffolds”), were fixed in 3% glutaraldehyde for 1 h and dehydrated with graded ethanol^64^. Then, samples were air-dried for 20 h and sputtered with 20 nm gold (Varian Vacuum Evaporator PS10E). An acceleration voltage of 15 kV was used for the SEM analysis (Hitachi TM3000 Tabletop Microscope).

### Antibiotic tolerance assay

Biofilm-containing scaffolds were incubated at 37 °C in control M9 media, or media supplemented with antibiotics (100 µg/mL ciprofloxacin or 200 µg/mL gentamicin, Sigma Aldrich). After 24 h, scaffolds were dried with sterile gauze and incubated during 4 h at 37 °C in darkness with 100 µL of XTT -PMS (phenazine methosulfate) solution (XTT 1 mg/mL + PMS 0.12 mg/mL in PBS, Sigma Aldrich), previously sterilized through a 0.22 µm syringe filter. Then, 200 µL of supernatants were taken and absorbance was measured at 492 nm using 650 nm as reference^65^, and the scaffolds were subjected to CLSM analysis following the protocol described above (“Confocal laser scanning microcopy (CLSM) and image processing”). Sterile scaffolds with M9 media were used as blank control.

### Scaffold implantation procedure

Surgeries were performed as described before, with slight modifications^66^. Briefly, 7–9 weeks old (19 to 25 g body weight) female C57/BL6 mice were anesthetized with ketamine (87.5 mg/kg) and xylazine (9 mg/kg), and hair was removed using a pet clipper and shaving cream (Veet®, Reckitt Benckiser). To prevent eye damage during surgery, an ophthalmic gel was applied (Nicotears®, Saval Laboratories), and 0.5 mL of saline solution was subcutaneously injected to avoid dehydration^66^. Afterwards, under biosafety cabinet, skin was sterilized with 2% chlorhexidine gluconate (Difem® Laboratories) and two 10-mm diameter bilateral full skin defects were surgically created in the back of the animal, using fine surgical scissors. Further, a titanized mesh of 13-mm diameter (TiMesh™, Pfm medical) was placed under the wound edges, and scaffolds were fixed by six single knots using non-absorbable surgical sutures (Ethilon 5/0, Johnson and Johnson). Finally, the implanted wounds were covered with a transparent dressing (Tegaderm™, 3 M). The day before surgery ciprofloxacin (30 mg/kg) was given as prophylaxis and injected during nine days as antibiotic therapy. For analgesic and antipyretic treatment, meloxicam (5 mg/kg) was administered before surgery and daily, after three days post-surgery. All animal experiments were performed according to protocols approved by the Ethics Committee of Universidad de Chile (19237—INT-UCH), and were conducted in accordance with the current Chilean legislation, the 3Rs guidelines from the UK National Centre for the Replacement Refinement and Reduction of Animals in Research, and the guidelines of the Care and Use of Laboratory Animals published by the US National Institute of Health. The study is reported according to the ARRIVE guideline 2.0.

### General health assessment

Animal health was daily supervised, following a general health score assessment sheet used for surgical interventions, that is based on Grimace Scale and NC3Rs guidelines^67^. This assigns 0 to 3 points for five parameters: (a) Weight loss ranging from < 5, 10, 20% of initial body weight; (b) General aspect of hair, posture, and secretions from eyes or ears; (c) Wound aspect, ranging from adequate hemostasia without edema to bleeding, inflammation or infection (yellow secretions); (d) Spontaneous behavior within the cage, from normal to diminished mobility and even self-mutilations; (e) Behavior in response to manipulation, from normal resistance to aggression or weakness with signs of pain. When two or more parameters have a score of 3, they increase to 4. Fifteen points was the humanitarian endpoint of experiment in case of septicemia, using an overdose of ketamine/xylazine intraperitoneal as euthanasia. Body temperature was daily measured with an infrared thermometer at the shaved back of the mice.

### Euthanasia and sample collection

After ten days of scaffold implantation, animals were euthanized by intraperitoneal overdose of ketamine/xylazine. Intracardiac blood was collected, clotted on ice for 1 h, centrifuged at 2000 *g* for 10 min at 4 °C, and 200 µL of blood serum was stored at − 80 °C for further analysis^68^. The remaining blood sample was resuspended in 0.5 mL PBS for CFU determination. Scaffolds were harvested and divided into quarters for protein extraction (stored at – 80 °C), histology (fixed in 4% formaldehyde for 24 h) and CFU counting (resuspended in 0.5 mL sterile PBS). Additionally, for histological analysis, a sample of the skin from the wound edge was also obtained and fixed in 4% formaldehyde for 24 h. Thymus, spleen, and lymph nodes were harvested, weighed, imaged using a stereomicroscope (Leica Biosystems), and fixed for histology.

### Pro-calcitonin quantitation

Pro-calcitonin was quantified in frozen serum samples using a Mouse Procalcitonin ELISA Kit (Novus biological) according to manufacturer’s instructions, preparing 1:2 dilutions of serum samples with the sample diluent included in the kit. For protein quantitation, a standard curve was performed to obtain the linear equation, which was further used to calculate the protein concentration of each serum sample.

### Cytokine bead assay

For protein extraction, frozen scaffolds were mechanically disrupted with pestles in 300 µL cold RIPA buffer with cOmplete™ Protease Inhibitor Cocktail (Roche) and sonicated for 1.5 min (30 s ON and OFF cycles, 40% amplitude). Then, 700 µL of RIPA buffer were added, and samples were incubated for 1 h at 4 °C and centrifuged for 20 min at 4 °C for supernatant collection. Total protein was quantified using Pierce™ BCA Protein Assay Kit (Thermo Scientific) following manufacturer’s instructions. For cytokine quantitation, protein extracts and serum samples from animals were processed as previously described using a BD™ CBA Mouse Inflammation Kit (BD Biosciences) and measured by fluorescence activated cell sorting (FACS)^68^.

### Histology and immunohistochemistry

Samples of the scaffolds were fixed in 4% paraformaldehyde for 24 h and stored at 4 °C in 70% ethanol, dehydrated, and embedded in Paraplast (Leica Biosystems). For morphological analysis, 5 μm sections were stained with Hematoxylin–Eosin (H&E) and Masson’s trichrome. Stained tissue sections were scanned at × 40 equivalent resolution using a slide scanner Aperio Versa (Leica Biosystems) and images were captured with Aperio ImageScope 12.4.6 software. Immunohistochemistry was performed according to previously published protocols^69^ using rabbit polyclonal anti-CD31 (PA5-16301 Invitrogen), 1:50 dilution; rabbit polyclonal anti-α-SMA (ab5694 Abcam), 1:1000 dilution; rabbit monoclonal anti-ki67 (MA5-14520 Invitrogen), 1:50 dilution. Bacterial visualization was performed using rabbit polyclonal anti-*Pseudomonas* (Abcam ab68538), 1:1000 dilution; rabbit polyclonal anti-*Staphylococcus* (Abcam ab20920), 1:1000 dilution; and rabbit polyclonal anti-*Enterococcus* (Abcam ab19980), 1:1000 dilution.

### Statistical analysis

All experiments were repeated in at least three independent assays, and data was expressed as mean ± SD or individual values accordingly. One-way or two-way analysis of variance (ANOVA) were used to evaluate one, two or more effects, using Tukey’s posterior or Sidak’s multiple comparison, to compare differences between groups. Unpaired t-test was used to compare differences between two groups. Differences were considered significant when *p* ≤ 0.05. Electronic laboratory notebook platform was not used.

## Supplementary Information

Supplementary Information.

## Data Availability

All data associated with this study are presented in this paper and can be shared with approved outside collaborators under a materials transfer agreement; requests should be sent to JTE, jte@uc.cl.
