# Supplementary material for: Development of an implantable three-dimensional model of a functional pathogenic multispecies biofilm to study infected wounds
Source: Sci Rep. 2022 Dec 17;12:21846. doi: 10.1038/s41598-022-25569-5 (PMC9759537; doi:10.1038/s41598-022-25569-5)
Supplement: Supplementary file 1 — Supplementary Information. [file 41598_2022_25569_MOESM1_ESM.docx]

**Supplementary information**

**
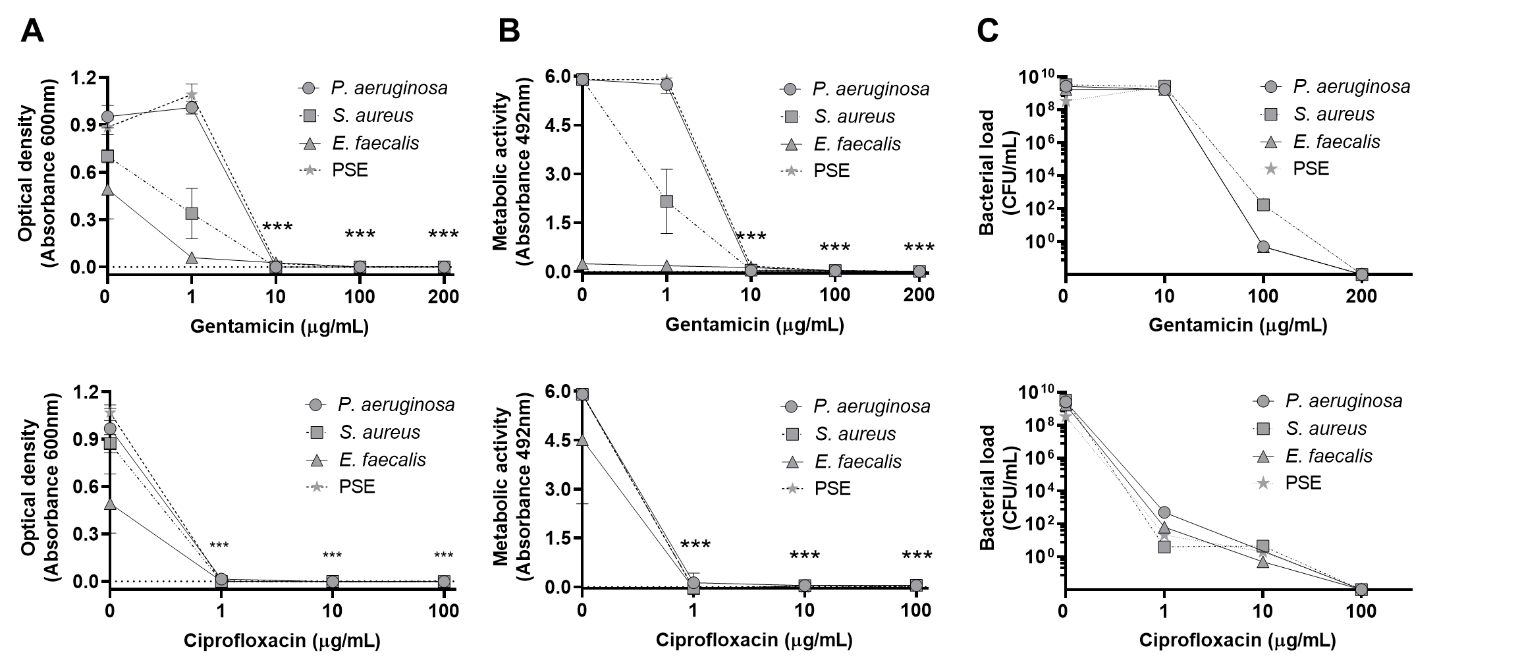
**

**Supplementary Figure 1. Antibiotic tolerance of planktonic bacteria.** Suspensions of 10^6^ cells/mL of each species (*P. aeruginosa*, *S. aureus* and *E. faecalis*) and the combination (PSE) were treated with antibiotics for 24 h. **A.** Optical density measurement after gentamicin or ciprofloxacin treatment, with a killing dose of 10 and 1 µg/mL respectively. **B.** XTT viability assay of bacterial suspensions for both antibiotics. **C.** Bacterial load quantitation of suspensions for both antibiotics. Values plotted are mean ± SD (N=2). Two-way ANOVA and Tukey’s posteriori comparison. ***p<0.001.


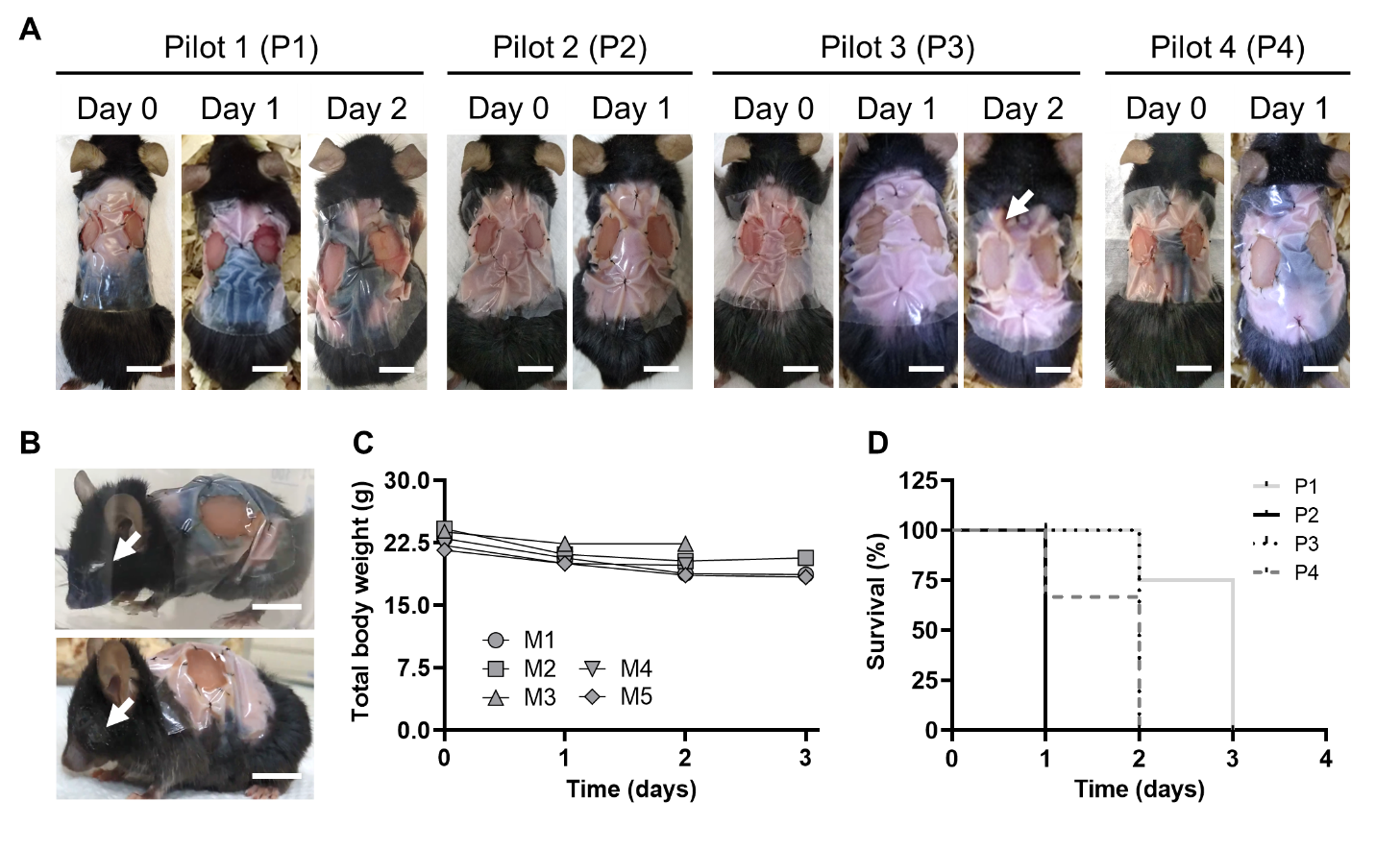


**Supplementary Figure 2. Experimental pilot studies to prevent septicemia outcome.** Female adult mice were implanted with biofilm-containing scaffolds incubated under different conditions. Pilot 1 (N=5): 10^8^ cells/mL for 3 days, Pilot 2 (N=2): 10^2^ cells/mL for 3 days, Pilot 3 (N=2): 10^2^ cells/mL for 1 day, Pilot 4 (N=2): 10^2^ cells/mL for 1 day with electrocauterization of skin bleeding during surgery. **A.** Representative images of daily supervision of animals from different pilot groups. Arrow indicates yellow color around the wound area. **B.** Representative images of mice’s aspect during septicemia, showing isolation, trembling and facial signs of pain. Arrows indicate secretion around the eyes. **C.** Total body weight of mice (M1 to M5) from Pilot 1 (N=5), showing the consistent weight loss up to 3 days after implantation. **D.** Survival curve of pilot groups indicating that most animals died between day 1 or 2 after biofilm implantation. Scale bars represents 10 mm. For C, values plotted are mean ± SD. For D, values plotted are mean values per pilot.


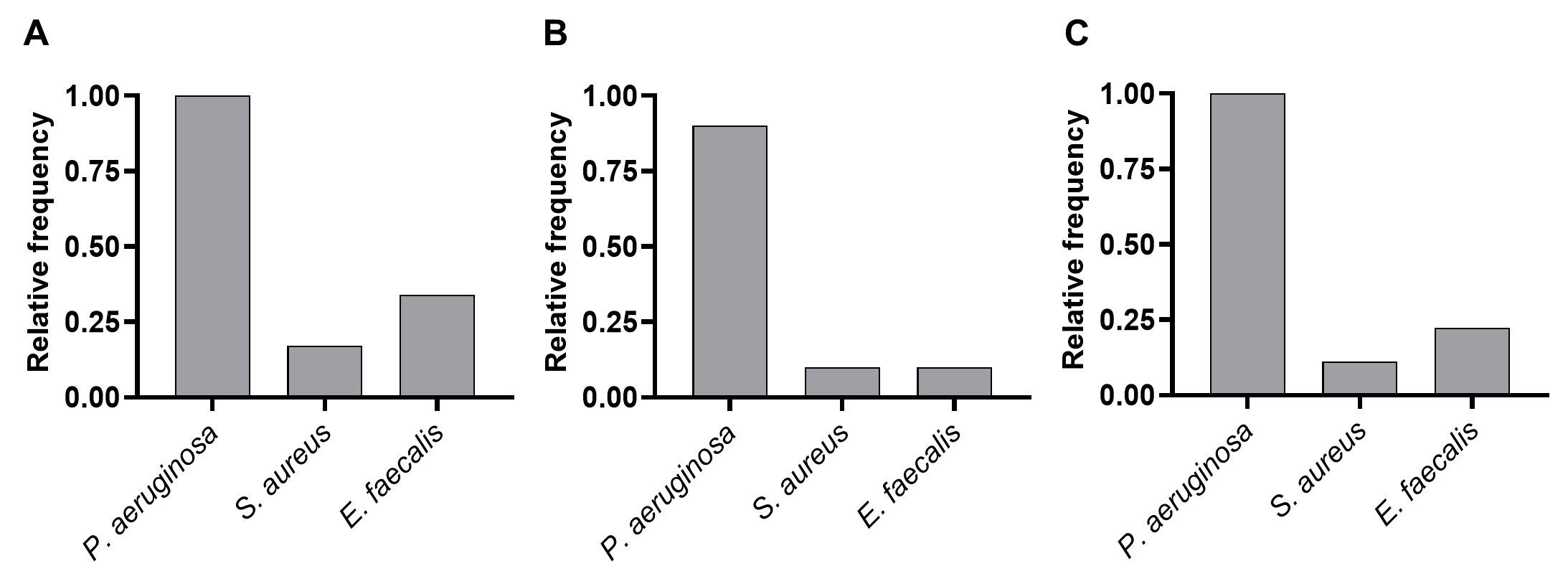


**Supplementary Figure 3. MALDI-TOF MS identification of bacterial species.** Samples of scaffolds or tissue were cultured to grow individual colonies, for identification of bacterial species from different experimental groups: **A.** Pre-implantation scaffolds incubated *in vitro* (N=11). **B.** Scaffolds after 10 days of implantation, with daily ciprofloxacin treatment of animals (N=21). **C.** Mice from pilot studies with septicemia outcome (N=9). Values plotted are means of frequency per bacterial species.
